# 

**Published:** 1877-11-20

**Authors:** 


ORIGINAL AND SELECTED ARTICLES—

Malarial Fevers—Cause and Treatment. By J. M. Lewis, M.D ..... 283
Teratology. By D. Newcomer, M.D ..... 285
Gelseminum. By I. J. M. Goss, M.A. M.D ..... 286
Hints on the Management of Labor. By A. H. Goelet, M.D ..... 288
Note on the Micrography and Chemistry of a "Pultaceous concretion" from the Tonsil. By John Vansant, M.D., Surgeon U. S. Marine Hospital Service ..... 289
Anthrax. By Jacob Van Valkenburgh, M D ..... 290
The Hygiene of the Eyes ..... 291
New Method of Reduction of Dislocation of the Hip. By S. J. Allen, M.D ..... 292

ABSTRACTS AND GLEANINGS—

Antiseptic Surgery, 294; Surgeon-Major Porter's Sawdust Pads, 295; Treatment of Croupous Pneumonia, 296; Collodion Flexile in Cases of Eczema, 297; A New Instrument for the Administration of Compressed Air, 298; The Treatment of Pulmonary Cavities, 299; Colocynth for Abdominal Pain, 300; How the Chinese make Eunuchs, 301; Mental Emotion as a Cause of Impotence, 301; Veratrum Viride Poisoning, 301; Chloral Drinker, 302; Cold and Sweating Feet, 302; Diagnosis of Hip Diseases in Children, 303; Masturbation, 303; Schiff on the Functions of the Spleen, 303; Abortive Power of Alcohol in Diptheria, 304; American Pharmaceutical Association, 304; When Not to Give Iron, 304; Cyanide of Zinc in Rheumatismal Neuralgia, 305; Patent Nostrums, 305; When Shall the Lying-in Woman Get Up ?, 305.

PRACTICAL NOTES AND FORMULÆ—

Hydrobromic Acid in Prescriptions, 306; Hydrobromic Acid in Cerebral Hyperæmia, 306; Hydrobromic Acid in Vertigo and Epilepsy, 306; Hydrobromic Acid in Nervous Dyspepsia, 307; Scraps of Practice, 307; How to Give Podophyllin, 308.

SCIENTIFIC ITEMS—

Mica, 308; Fire, 308; Pharaoh's Serpents' Eggs, 308; Lavœsium, 308.

EDITORIAL AND MISCELLANEOUS—

Remittances for 1878, 309; Our Journal, 309; Dr. C. G. Polk and the "Hospital Gazette," 309; Death of Dr. Paul F. Eve, 309; Sugar-Coated Pills, 310; U. S. Signal Service, 310; Pemberton, Samuels & Reynolds, 311; University of Michigan, 311; The Christian Index, 311; College Embroglio, 311; Home for Opium Habitues, 311; Bound Volumes of the Record, 311; Fresh Meat Cure, 311; Nashville Medical College, 311; Compressed Pills, 311; Receipted for 1877, 311; Advertisements.

BOOK NOTICES.

Transactions of the American Medical Association, 310; Wood's Physician's Vade Mecum, and Visiting List, 310; Personal Appearance and the Culture of Beauty, 310; New Pessaries, 310; The Columbia Hospital and Lying-in Asylum, 310.

